# Supplementary material for: Modeling glioblastoma heterogeneity as a dynamic network of cell states
Source: Mol Syst Biol. 2021 Sep 16;17(9):e10105. doi: 10.15252/msb.202010105 (PMC8444284; doi:10.15252/msb.202010105)
Supplement: Supplementary file 6 — Source Data for Figure 5 [file MSB-17-e10105-s004.zip › Figure5A_sourcedata/GSEA_3071/nosigdb_stateII.GseaPreranked.1622795151171/DOROSZKO_U3180MG_CLUSTER_3.html]

Details for gene set DOROSZKO\_U3180MG\_CLUSTER\_3[GSEA]

|  || Dataset | state23071 |
| Phenotype | NoPhenotypeAvailable |
| Upregulated in class | na\_pos |
| GeneSet | DOROSZKO\_U3180MG\_CLUSTER\_3 |
| Enrichment Score (ES) | 0.7163794 |
| Normalized Enrichment Score (NES) | 5.44272 |
| Nominal p-value | 0.0 |
| FDR q-value | 0.0 |
| FWER p-Value | 0.0 |
Table: GSEA Results Summary

  

Fig 1: Enrichment plot: DOROSZKO\_U3180MG\_CLUSTER\_3      
 Profile of the Running ES Score & Positions of GeneSet Members on the Rank Ordered List

  

| PROBE | GENE SYMBOL | GENE\_TITLE | RANK IN GENE LIST | RANK METRIC SCORE | RUNNING ES | CORE ENRICHMENT || 1 | HIST1H1D |  |  | 0 | 2.323 | 0.0078 | Yes |
| 2 | CLSPN |  |  | 1 | 1.984 | 0.0145 | Yes |
| 3 | HIST1H4C |  |  | 2 | 1.902 | 0.0210 | Yes |
| 4 | RRM2 |  |  | 3 | 1.857 | 0.0272 | Yes |
| 5 | TYMS |  |  | 8 | 1.665 | 0.0318 | Yes |
| 6 | MCM10 |  |  | 9 | 1.654 | 0.0374 | Yes |
| 7 | PCLAF |  |  | 10 | 1.638 | 0.0429 | Yes |
| 8 | CDC6 |  |  | 11 | 1.631 | 0.0484 | Yes |
| 9 | CDK1 |  |  | 14 | 1.540 | 0.0531 | Yes |
| 10 | DIAPH3 |  |  | 15 | 1.531 | 0.0582 | Yes |
| 11 | ZWINT |  |  | 16 | 1.510 | 0.0633 | Yes |
| 12 | HJURP |  |  | 17 | 1.490 | 0.0684 | Yes |
| 13 | ATAD5 |  |  | 18 | 1.469 | 0.0733 | Yes |
| 14 | FEN1 |  |  | 19 | 1.464 | 0.0783 | Yes |
| 15 | BRCA2 |  |  | 22 | 1.445 | 0.0826 | Yes |
| 16 | UBE2C |  |  | 23 | 1.438 | 0.0875 | Yes |
| 17 | CENPU |  |  | 24 | 1.416 | 0.0922 | Yes |
| 18 | RAD51AP1 |  |  | 25 | 1.384 | 0.0969 | Yes |
| 19 | CENPK |  |  | 26 | 1.382 | 0.1016 | Yes |
| 20 | CDCA4 |  |  | 27 | 1.376 | 0.1062 | Yes |
| 21 | MYBL2 |  |  | 28 | 1.362 | 0.1108 | Yes |
| 22 | FAM111B |  |  | 29 | 1.361 | 0.1154 | Yes |
| 23 | FBXO5 |  |  | 30 | 1.359 | 0.1200 | Yes |
| 24 | USP1 |  |  | 31 | 1.357 | 0.1246 | Yes |
| 25 | TCF19 |  |  | 32 | 1.331 | 0.1291 | Yes |
| 26 | DTL |  |  | 33 | 1.330 | 0.1336 | Yes |
| 27 | SLC43A3 |  |  | 34 | 1.323 | 0.1380 | Yes |
| 28 | PSMC3IP |  |  | 36 | 1.322 | 0.1422 | Yes |
| 29 | RRM1 |  |  | 37 | 1.310 | 0.1467 | Yes |
| 30 | ORC6 |  |  | 39 | 1.308 | 0.1508 | Yes |
| 31 | GINS2 |  |  | 40 | 1.306 | 0.1552 | Yes |
| 32 | CDCA5 |  |  | 41 | 1.289 | 0.1596 | Yes |
| 33 | TK1 |  |  | 42 | 1.281 | 0.1639 | Yes |
| 34 | AURKB |  |  | 44 | 1.276 | 0.1679 | Yes |
| 35 | MCM7 |  |  | 45 | 1.273 | 0.1722 | Yes |
| 36 | HELLS |  |  | 46 | 1.259 | 0.1765 | Yes |
| 37 | MCM8 |  |  | 48 | 1.253 | 0.1804 | Yes |
| 38 | BRCA1 |  |  | 49 | 1.249 | 0.1847 | Yes |
| 39 | CDT1 |  |  | 51 | 1.244 | 0.1886 | Yes |
| 40 | GINS1 |  |  | 52 | 1.239 | 0.1928 | Yes |
| 41 | CCNA2 |  |  | 53 | 1.230 | 0.1969 | Yes |
| 42 | DNAJC9 |  |  | 56 | 1.217 | 0.2005 | Yes |
| 43 | SKA3 |  |  | 57 | 1.215 | 0.2046 | Yes |
| 44 | PCNA |  |  | 58 | 1.214 | 0.2087 | Yes |
| 45 | CDC45 |  |  | 59 | 1.212 | 0.2128 | Yes |
| 46 | MAD2L1 |  |  | 61 | 1.199 | 0.2166 | Yes |
| 47 | UBE2T |  |  | 62 | 1.192 | 0.2206 | Yes |
| 48 | ATAD2 |  |  | 63 | 1.189 | 0.2246 | Yes |
| 49 | PRIM1 |  |  | 65 | 1.170 | 0.2283 | Yes |
| 50 | MSH2 |  |  | 66 | 1.163 | 0.2322 | Yes |
| 51 | RFC3 |  |  | 67 | 1.157 | 0.2361 | Yes |
| 52 | SPC25 |  |  | 68 | 1.156 | 0.2400 | Yes |
| 53 | MELK |  |  | 69 | 1.145 | 0.2439 | Yes |
| 54 | POLD3 |  |  | 70 | 1.143 | 0.2478 | Yes |
| 55 | WDHD1 |  |  | 71 | 1.133 | 0.2516 | Yes |
| 56 | TOP2A |  |  | 72 | 1.132 | 0.2554 | Yes |
| 57 | KIF2C |  |  | 73 | 1.132 | 0.2592 | Yes |
| 58 | MCM5 |  |  | 74 | 1.128 | 0.2630 | Yes |
| 59 | DSN1 |  |  | 75 | 1.122 | 0.2668 | Yes |
| 60 | CCNE2 |  |  | 76 | 1.121 | 0.2706 | Yes |
| 61 | IDH2 |  |  | 78 | 1.117 | 0.2741 | Yes |
| 62 | UHRF1 |  |  | 79 | 1.113 | 0.2779 | Yes |
| 63 | FAM111A |  |  | 80 | 1.112 | 0.2816 | Yes |
| 64 | MCM4 |  |  | 81 | 1.110 | 0.2854 | Yes |
| 65 | RBL1 |  |  | 82 | 1.110 | 0.2891 | Yes |
| 66 | GMNN |  |  | 83 | 1.108 | 0.2929 | Yes |
| 67 | RBBP8 |  |  | 86 | 1.100 | 0.2960 | Yes |
| 68 | KIFC1 |  |  | 88 | 1.094 | 0.2995 | Yes |
| 69 | HIST1H1E |  |  | 90 | 1.088 | 0.3029 | Yes |
| 70 | SKA1 |  |  | 91 | 1.078 | 0.3065 | Yes |
| 71 | XRCC2 |  |  | 92 | 1.076 | 0.3101 | Yes |
| 72 | VRK1 |  |  | 93 | 1.074 | 0.3138 | Yes |
| 73 | NDC80 |  |  | 94 | 1.074 | 0.3174 | Yes |
| 74 | POLA2 |  |  | 95 | 1.068 | 0.3210 | Yes |
| 75 | MASTL |  |  | 96 | 1.063 | 0.3246 | Yes |
| 76 | LRR1 |  |  | 98 | 1.060 | 0.3279 | Yes |
| 77 | TMPO |  |  | 99 | 1.056 | 0.3315 | Yes |
| 78 | NCAPG |  |  | 100 | 1.056 | 0.3350 | Yes |
| 79 | DHFR |  |  | 101 | 1.051 | 0.3386 | Yes |
| 80 | MYBL1 |  |  | 102 | 1.043 | 0.3421 | Yes |
| 81 | MND1 |  |  | 104 | 1.036 | 0.3453 | Yes |
| 82 | MCM3 |  |  | 105 | 1.035 | 0.3488 | Yes |
| 83 | MKI67 |  |  | 107 | 1.031 | 0.3520 | Yes |
| 84 | FANCA |  |  | 109 | 1.029 | 0.3552 | Yes |
| 85 | BRIP1 |  |  | 110 | 1.029 | 0.3587 | Yes |
| 86 | TPX2 |  |  | 111 | 1.025 | 0.3622 | Yes |
| 87 | RFC2 |  |  | 112 | 1.022 | 0.3656 | Yes |
| 88 | TRIP13 |  |  | 114 | 1.014 | 0.3688 | Yes |
| 89 | NCAPH |  |  | 116 | 1.013 | 0.3719 | Yes |
| 90 | NCAPD3 |  |  | 117 | 1.013 | 0.3754 | Yes |
| 91 | DUT |  |  | 118 | 1.009 | 0.3788 | Yes |
| 92 | ASF1B |  |  | 120 | 1.007 | 0.3819 | Yes |
| 93 | SMC2 |  |  | 123 | 1.005 | 0.3847 | Yes |
| 94 | NUF2 |  |  | 125 | 1.004 | 0.3879 | Yes |
| 95 | DEPDC1 |  |  | 126 | 0.999 | 0.3912 | Yes |
| 96 | CHAF1A |  |  | 127 | 0.999 | 0.3946 | Yes |
| 97 | CKS1B |  |  | 128 | 0.999 | 0.3980 | Yes |
| 98 | TACC3 |  |  | 130 | 0.992 | 0.4011 | Yes |
| 99 | MGME1 |  |  | 135 | 0.988 | 0.4033 | Yes |
| 100 | PFKP |  |  | 136 | 0.988 | 0.4067 | Yes |
| 101 | CDK4 |  |  | 137 | 0.986 | 0.4100 | Yes |
| 102 | FANCI |  |  | 138 | 0.984 | 0.4133 | Yes |
| 103 | WDR76 |  |  | 142 | 0.975 | 0.4158 | Yes |
| 104 | ESCO2 |  |  | 144 | 0.971 | 0.4188 | Yes |
| 105 | EXO1 |  |  | 145 | 0.969 | 0.4221 | Yes |
| 106 | PLP2 |  |  | 147 | 0.962 | 0.4251 | Yes |
| 107 | CKAP2L |  |  | 148 | 0.959 | 0.4283 | Yes |
| 108 | RANBP1 |  |  | 149 | 0.958 | 0.4315 | Yes |
| 109 | E2F1 |  |  | 153 | 0.951 | 0.4339 | Yes |
| 110 | DSCC1 |  |  | 155 | 0.946 | 0.4369 | Yes |
| 111 | NCAPG2 |  |  | 159 | 0.937 | 0.4392 | Yes |
| 112 | GGCT |  |  | 160 | 0.937 | 0.4424 | Yes |
| 113 | KIF23 |  |  | 161 | 0.936 | 0.4455 | Yes |
| 114 | CTPS1 |  |  | 162 | 0.934 | 0.4487 | Yes |
| 115 | DONSON |  |  | 163 | 0.933 | 0.4518 | Yes |
| 116 | SHCBP1 |  |  | 164 | 0.933 | 0.4550 | Yes |
| 117 | RTKN2 |  |  | 166 | 0.931 | 0.4579 | Yes |
| 118 | GTSE1 |  |  | 167 | 0.929 | 0.4610 | Yes |
| 119 | RPA2 |  |  | 169 | 0.929 | 0.4639 | Yes |
| 120 | CDCA2 |  |  | 171 | 0.922 | 0.4667 | Yes |
| 121 | MTHFD1 |  |  | 173 | 0.914 | 0.4695 | Yes |
| 122 | MCM6 |  |  | 176 | 0.906 | 0.4720 | Yes |
| 123 | CENPM |  |  | 178 | 0.901 | 0.4748 | Yes |
| 124 | KPNB1 |  |  | 186 | 0.891 | 0.4759 | Yes |
| 125 | SAC3D1 |  |  | 188 | 0.887 | 0.4787 | Yes |
| 126 | C21orf58 |  |  | 201 | 0.873 | 0.4784 | Yes |
| 127 | NCAPH2 |  |  | 207 | 0.868 | 0.4800 | Yes |
| 128 | TFDP1 |  |  | 209 | 0.866 | 0.4826 | Yes |
| 129 | CSE1L |  |  | 210 | 0.866 | 0.4855 | Yes |
| 130 | PRIM2 |  |  | 212 | 0.865 | 0.4882 | Yes |
| 131 | H2AFZ |  |  | 213 | 0.864 | 0.4911 | Yes |
| 132 | PBK |  |  | 214 | 0.864 | 0.4940 | Yes |
| 133 | CEP152 |  |  | 217 | 0.860 | 0.4964 | Yes |
| 134 | CDCA3 |  |  | 219 | 0.858 | 0.4990 | Yes |
| 135 | CENPH |  |  | 221 | 0.855 | 0.5016 | Yes |
| 136 | RFC5 |  |  | 222 | 0.851 | 0.5045 | Yes |
| 137 | BUB1B |  |  | 223 | 0.851 | 0.5074 | Yes |
| 138 | CHAF1B |  |  | 229 | 0.845 | 0.5089 | Yes |
| 139 | CCDC15 |  |  | 230 | 0.845 | 0.5117 | Yes |
| 140 | ASPM |  |  | 231 | 0.842 | 0.5146 | Yes |
| 141 | EZH2 |  |  | 233 | 0.839 | 0.5171 | Yes |
| 142 | SLF1 |  |  | 237 | 0.834 | 0.5191 | Yes |
| 143 | HMGA1 |  |  | 238 | 0.834 | 0.5220 | Yes |
| 144 | MIS18A |  |  | 239 | 0.833 | 0.5248 | Yes |
| 145 | DBF4 |  |  | 240 | 0.832 | 0.5276 | Yes |
| 146 | RACGAP1 |  |  | 241 | 0.831 | 0.5304 | Yes |
| 147 | PRC1 |  |  | 242 | 0.830 | 0.5332 | Yes |
| 148 | TUBB6 |  |  | 244 | 0.828 | 0.5357 | Yes |
| 149 | C19orf48 |  |  | 248 | 0.826 | 0.5377 | Yes |
| 150 | LMNB1 |  |  | 251 | 0.824 | 0.5399 | Yes |
| 151 | CHEK1 |  |  | 252 | 0.822 | 0.5427 | Yes |
| 152 | DNMT1 |  |  | 265 | 0.792 | 0.5422 | Yes |
| 153 | TUBB4B |  |  | 271 | 0.784 | 0.5435 | Yes |
| 154 | HMGB2 |  |  | 276 | 0.780 | 0.5450 | Yes |
| 155 | TUBA1C |  |  | 277 | 0.779 | 0.5476 | Yes |
| 156 | SMC4 |  |  | 279 | 0.777 | 0.5500 | Yes |
| 157 | HSPB11 |  |  | 283 | 0.775 | 0.5518 | Yes |
| 158 | TEX30 |  |  | 287 | 0.774 | 0.5536 | Yes |
| 159 | BARD1 |  |  | 295 | 0.760 | 0.5543 | Yes |
| 160 | POLE3 |  |  | 297 | 0.758 | 0.5566 | Yes |
| 161 | DEK |  |  | 302 | 0.754 | 0.5580 | Yes |
| 162 | MMS22L |  |  | 306 | 0.747 | 0.5598 | Yes |
| 163 | KIF4A |  |  | 309 | 0.746 | 0.5617 | Yes |
| 164 | COPS3 |  |  | 310 | 0.746 | 0.5643 | Yes |
| 165 | CKS2 |  |  | 312 | 0.745 | 0.5665 | Yes |
| 166 | ALYREF |  |  | 315 | 0.741 | 0.5685 | Yes |
| 167 | SLBP |  |  | 318 | 0.740 | 0.5704 | Yes |
| 168 | CENPN |  |  | 320 | 0.737 | 0.5726 | Yes |
| 169 | PKMYT1 |  |  | 322 | 0.736 | 0.5749 | Yes |
| 170 | ANP32B |  |  | 325 | 0.734 | 0.5768 | Yes |
| 171 | NUP85 |  |  | 328 | 0.729 | 0.5787 | Yes |
| 172 | CENPW |  |  | 330 | 0.727 | 0.5809 | Yes |
| 173 | WDR34 |  |  | 333 | 0.724 | 0.5828 | Yes |
| 174 | SPC24 |  |  | 340 | 0.719 | 0.5836 | Yes |
| 175 | LIG1 |  |  | 341 | 0.718 | 0.5860 | Yes |
| 176 | RPA1 |  |  | 343 | 0.715 | 0.5882 | Yes |
| 177 | ARHGAP11A |  |  | 346 | 0.712 | 0.5901 | Yes |
| 178 | SPATA33 |  |  | 347 | 0.712 | 0.5925 | Yes |
| 179 | LMNB2 |  |  | 352 | 0.709 | 0.5938 | Yes |
| 180 | DTYMK |  |  | 364 | 0.701 | 0.5932 | Yes |
| 181 | PSMG1 |  |  | 372 | 0.696 | 0.5936 | Yes |
| 182 | CENPJ |  |  | 376 | 0.695 | 0.5952 | Yes |
| 183 | TMEM106C |  |  | 377 | 0.694 | 0.5975 | Yes |
| 184 | E2F8 |  |  | 379 | 0.691 | 0.5996 | Yes |
| 185 | SIVA1 |  |  | 380 | 0.690 | 0.6019 | Yes |
| 186 | CHAC2 |  |  | 381 | 0.690 | 0.6042 | Yes |
| 187 | PARPBP |  |  | 382 | 0.688 | 0.6066 | Yes |
| 188 | JPT2 |  |  | 384 | 0.687 | 0.6086 | Yes |
| 189 | RFC4 |  |  | 385 | 0.686 | 0.6109 | Yes |
| 190 | MTFR2 |  |  | 386 | 0.686 | 0.6132 | Yes |
| 191 | CCND3 |  |  | 390 | 0.683 | 0.6147 | Yes |
| 192 | SUPT16H |  |  | 394 | 0.682 | 0.6162 | Yes |
| 193 | TMEM97 |  |  | 403 | 0.676 | 0.6164 | Yes |
| 194 | KIF15 |  |  | 404 | 0.676 | 0.6187 | Yes |
| 195 | CTNNAL1 |  |  | 405 | 0.676 | 0.6209 | Yes |
| 196 | NUDC |  |  | 407 | 0.673 | 0.6229 | Yes |
| 197 | ZNF367 |  |  | 408 | 0.673 | 0.6252 | Yes |
| 198 | CCDC34 |  |  | 411 | 0.672 | 0.6269 | Yes |
| 199 | PA2G4 |  |  | 412 | 0.672 | 0.6292 | Yes |
| 200 | TEDC1 |  |  | 414 | 0.670 | 0.6312 | Yes |
| 201 | SGO2 |  |  | 415 | 0.670 | 0.6335 | Yes |
| 202 | FAF1 |  |  | 416 | 0.669 | 0.6357 | Yes |
| 203 | GINS4 |  |  | 417 | 0.669 | 0.6380 | Yes |
| 204 | SAE1 |  |  | 418 | 0.669 | 0.6402 | Yes |
| 205 | KPNA2 |  |  | 420 | 0.669 | 0.6422 | Yes |
| 206 | FOXM1 |  |  | 423 | 0.667 | 0.6439 | Yes |
| 207 | SMC3 |  |  | 425 | 0.667 | 0.6459 | Yes |
| 208 | PHGDH |  |  | 429 | 0.664 | 0.6474 | Yes |
| 209 | HNRNPAB |  |  | 435 | 0.659 | 0.6482 | Yes |
| 210 | ZWILCH |  |  | 436 | 0.659 | 0.6505 | Yes |
| 211 | BUB3 |  |  | 441 | 0.657 | 0.6516 | Yes |
| 212 | ACAT2 |  |  | 446 | 0.653 | 0.6527 | Yes |
| 213 | PARP2 |  |  | 451 | 0.650 | 0.6538 | Yes |
| 214 | RAD54L |  |  | 456 | 0.648 | 0.6549 | Yes |
| 215 | NUDT15 |  |  | 461 | 0.646 | 0.6561 | Yes |
| 216 | RAD18 |  |  | 465 | 0.642 | 0.6574 | Yes |
| 217 | SNRPB |  |  | 467 | 0.642 | 0.6593 | Yes |
| 218 | ARPC5L |  |  | 471 | 0.640 | 0.6607 | Yes |
| 219 | DCTPP1 |  |  | 477 | 0.638 | 0.6615 | Yes |
| 220 | SSRP1 |  |  | 478 | 0.638 | 0.6636 | Yes |
| 221 | CKLF |  |  | 482 | 0.637 | 0.6650 | Yes |
| 222 | FADS1 |  |  | 498 | 0.629 | 0.6630 | Yes |
| 223 | POP7 |  |  | 502 | 0.628 | 0.6644 | Yes |
| 224 | KNL1 |  |  | 504 | 0.628 | 0.6662 | Yes |
| 225 | BIRC5 |  |  | 505 | 0.628 | 0.6683 | Yes |
| 226 | MRPL37 |  |  | 513 | 0.623 | 0.6685 | Yes |
| 227 | PANK2 |  |  | 519 | 0.618 | 0.6693 | Yes |
| 228 | SNRPD1 |  |  | 523 | 0.615 | 0.6706 | Yes |
| 229 | HAUS8 |  |  | 527 | 0.613 | 0.6718 | Yes |
| 230 | SGO1 |  |  | 528 | 0.612 | 0.6739 | Yes |
| 231 | CDK2 |  |  | 530 | 0.612 | 0.6757 | Yes |
| 232 | RPA3 |  |  | 532 | 0.611 | 0.6775 | Yes |
| 233 | C5orf34 |  |  | 533 | 0.611 | 0.6795 | Yes |
| 234 | BTG3 |  |  | 540 | 0.607 | 0.6800 | Yes |
| 235 | DDX39A |  |  | 542 | 0.605 | 0.6817 | Yes |
| 236 | FUCA2 |  |  | 543 | 0.605 | 0.6838 | Yes |
| 237 | RCC1 |  |  | 549 | 0.603 | 0.6845 | Yes |
| 238 | CENPX |  |  | 561 | 0.595 | 0.6835 | Yes |
| 239 | CENPQ |  |  | 569 | 0.590 | 0.6836 | Yes |
| 240 | MIS18BP1 |  |  | 574 | 0.588 | 0.6845 | Yes |
| 241 | CENPF |  |  | 575 | 0.588 | 0.6865 | Yes |
| 242 | SCCPDH |  |  | 582 | 0.586 | 0.6869 | Yes |
| 243 | TIMELESS |  |  | 591 | 0.583 | 0.6867 | Yes |
| 244 | CEP135 |  |  | 598 | 0.578 | 0.6870 | Yes |
| 245 | EXOSC8 |  |  | 604 | 0.576 | 0.6876 | Yes |
| 246 | SMS |  |  | 608 | 0.575 | 0.6887 | Yes |
| 247 | SRPK1 |  |  | 610 | 0.574 | 0.6904 | Yes |
| 248 | MSH6 |  |  | 611 | 0.574 | 0.6924 | Yes |
| 249 | CACYBP |  |  | 613 | 0.573 | 0.6940 | Yes |
| 250 | TUBG1 |  |  | 617 | 0.570 | 0.6951 | Yes |
| 251 | SMC1A |  |  | 618 | 0.570 | 0.6971 | Yes |
| 252 | CKAP2 |  |  | 619 | 0.569 | 0.6990 | Yes |
| 253 | HAUS2 |  |  | 621 | 0.569 | 0.7006 | Yes |
| 254 | FARSB |  |  | 625 | 0.568 | 0.7017 | Yes |
| 255 | HAT1 |  |  | 632 | 0.565 | 0.7020 | Yes |
| 256 | PPIF |  |  | 639 | 0.560 | 0.7023 | Yes |
| 257 | COMMD4 |  |  | 644 | 0.554 | 0.7031 | Yes |
| 258 | HAUS1 |  |  | 649 | 0.552 | 0.7039 | Yes |
| 259 | YEATS4 |  |  | 652 | 0.550 | 0.7052 | Yes |
| 260 | NUSAP1 |  |  | 654 | 0.550 | 0.7068 | Yes |
| 261 | DCK |  |  | 661 | 0.548 | 0.7070 | Yes |
| 262 | CCT5 |  |  | 662 | 0.548 | 0.7089 | Yes |
| 263 | H2AFX |  |  | 671 | 0.544 | 0.7085 | Yes |
| 264 | MNS1 |  |  | 672 | 0.543 | 0.7104 | Yes |
| 265 | TUBB |  |  | 678 | 0.538 | 0.7109 | Yes |
| 266 | PRKDC |  |  | 681 | 0.537 | 0.7121 | Yes |
| 267 | MRPL17 |  |  | 682 | 0.537 | 0.7139 | Yes |
| 268 | DEGS1 |  |  | 688 | 0.536 | 0.7144 | Yes |
| 269 | PPIH |  |  | 695 | 0.533 | 0.7146 | Yes |
| 270 | CIP2A |  |  | 696 | 0.533 | 0.7164 | Yes |
| 271 | BAZ1B |  |  | 721 | 0.525 | 0.7117 | No |
| 272 | FANCG |  |  | 723 | 0.525 | 0.7132 | No |
| 273 | MCM2 |  |  | 734 | 0.521 | 0.7122 | No |
| 274 | PHF19 |  |  | 745 | 0.518 | 0.7113 | No |
| 275 | UNG |  |  | 750 | 0.516 | 0.7120 | No |
| 276 | KDELC2 |  |  | 754 | 0.515 | 0.7129 | No |
| 277 | SNX5 |  |  | 762 | 0.512 | 0.7127 | No |
| 278 | AUNIP |  |  | 770 | 0.508 | 0.7126 | No |
| 279 | CMC2 |  |  | 785 | 0.503 | 0.7105 | No |
| 280 | HACD3 |  |  | 800 | 0.498 | 0.7084 | No |
| 281 | EIF4EBP1 |  |  | 805 | 0.496 | 0.7090 | No |
| 282 | SUMO3 |  |  | 825 | 0.490 | 0.7055 | No |
| 283 | ACYP1 |  |  | 834 | 0.489 | 0.7050 | No |
| 284 | EEF1E1 |  |  | 840 | 0.488 | 0.7053 | No |
| 285 | UBR7 |  |  | 857 | 0.483 | 0.7026 | No |
| 286 | AP2S1 |  |  | 871 | 0.479 | 0.7007 | No |
| 287 | IKBIP |  |  | 876 | 0.477 | 0.7013 | No |
| 288 | ARL6IP6 |  |  | 880 | 0.476 | 0.7021 | No |
| 289 | DERA |  |  | 886 | 0.474 | 0.7023 | No |
| 290 | FAM161A |  |  | 890 | 0.473 | 0.7031 | No |
| 291 | CISD2 |  |  | 891 | 0.473 | 0.7047 | No |
| 292 | SUZ12 |  |  | 894 | 0.472 | 0.7058 | No |
| 293 | DEPDC1B |  |  | 906 | 0.470 | 0.7044 | No |
| 294 | RAD1 |  |  | 926 | 0.464 | 0.7008 | No |
| 295 | SVIP |  |  | 931 | 0.463 | 0.7013 | No |
| 296 | KDELR2 |  |  | 933 | 0.463 | 0.7026 | No |
| 297 | MCMBP |  |  | 934 | 0.462 | 0.7042 | No |
| 298 | ITGB3BP |  |  | 935 | 0.462 | 0.7057 | No |
| 299 | HIRIP3 |  |  | 936 | 0.462 | 0.7073 | No |
| 300 | GPN3 |  |  | 953 | 0.458 | 0.7045 | No |
| 301 | RAD51C |  |  | 967 | 0.453 | 0.7025 | No |
| 302 | CDC7 |  |  | 971 | 0.451 | 0.7033 | No |
| 303 | KIF22 |  |  | 975 | 0.450 | 0.7040 | No |
| 304 | FANCD2 |  |  | 976 | 0.450 | 0.7055 | No |
| 305 | TEDC2 |  |  | 979 | 0.449 | 0.7065 | No |
| 306 | SF3B4 |  |  | 995 | 0.446 | 0.7039 | No |
| 307 | CCT4 |  |  | 999 | 0.445 | 0.7046 | No |
| 308 | NR2C2AP |  |  | 1013 | 0.440 | 0.7026 | No |
| 309 | CASP8AP2 |  |  | 1025 | 0.436 | 0.7011 | No |
| 310 | PDCD5 |  |  | 1051 | 0.428 | 0.6958 | No |
| 311 | CEP57 |  |  | 1055 | 0.426 | 0.6964 | No |
| 312 | NUDT21 |  |  | 1076 | 0.422 | 0.6925 | No |
| 313 | POC1A |  |  | 1080 | 0.421 | 0.6931 | No |
| 314 | SAAL1 |  |  | 1098 | 0.417 | 0.6899 | No |
| 315 | CCT2 |  |  | 1108 | 0.415 | 0.6889 | No |
| 316 | HNRNPF |  |  | 1128 | 0.412 | 0.6852 | No |
| 317 | UBA2 |  |  | 1131 | 0.412 | 0.6860 | No |
| 318 | CBX5 |  |  | 1132 | 0.412 | 0.6874 | No |
| 319 | SNRPF |  |  | 1150 | 0.407 | 0.6842 | No |
| 320 | TPRKB |  |  | 1168 | 0.403 | 0.6810 | No |
| 321 | HNRNPD |  |  | 1177 | 0.401 | 0.6802 | No |
| 322 | DDB2 |  |  | 1189 | 0.399 | 0.6785 | No |
| 323 | RAB23 |  |  | 1190 | 0.399 | 0.6799 | No |
| 324 | CHTF18 |  |  | 1192 | 0.398 | 0.6810 | No |
| 325 | FBXO4 |  |  | 1210 | 0.394 | 0.6777 | No |
| 326 | MRM2 |  |  | 1220 | 0.393 | 0.6766 | No |
| 327 | VPS29 |  |  | 1234 | 0.390 | 0.6744 | No |
| 328 | PSMC3 |  |  | 1241 | 0.389 | 0.6741 | No |
| 329 | RMI2 |  |  | 1279 | 0.381 | 0.6654 | No |
| 330 | POLE4 |  |  | 1281 | 0.381 | 0.6665 | No |
| 331 | RECQL4 |  |  | 1285 | 0.380 | 0.6669 | No |
| 332 | ABHD3 |  |  | 1288 | 0.380 | 0.6677 | No |
| 333 | IER3IP1 |  |  | 1299 | 0.378 | 0.6663 | No |
| 334 | SNRPD3 |  |  | 1303 | 0.377 | 0.6667 | No |
| 335 | EIF4A3 |  |  | 1332 | 0.372 | 0.6604 | No |
| 336 | ANP32E |  |  | 1334 | 0.371 | 0.6614 | No |
| 337 | MAGOHB |  |  | 1336 | 0.369 | 0.6624 | No |
| 338 | NME1 |  |  | 1343 | 0.368 | 0.6620 | No |
| 339 | FDPS |  |  | 1348 | 0.367 | 0.6622 | No |
| 340 | SHMT2 |  |  | 1361 | 0.366 | 0.6602 | No |
| 341 | RALY |  |  | 1368 | 0.362 | 0.6598 | No |
| 342 | KLHL23 |  |  | 1369 | 0.362 | 0.6610 | No |
| 343 | AK2 |  |  | 1378 | 0.360 | 0.6601 | No |
| 344 | ITGB1BP1 |  |  | 1382 | 0.360 | 0.6605 | No |
| 345 | SLC20A1 |  |  | 1400 | 0.357 | 0.6571 | No |
| 346 | PARP1 |  |  | 1431 | 0.351 | 0.6502 | No |
| 347 | IMMT |  |  | 1436 | 0.350 | 0.6503 | No |
| 348 | AHCY |  |  | 1471 | 0.344 | 0.6423 | No |
| 349 | SRR |  |  | 1482 | 0.342 | 0.6408 | No |
| 350 | NUDT5 |  |  | 1486 | 0.342 | 0.6411 | No |
| 351 | CBR3 |  |  | 1522 | 0.336 | 0.6328 | No |
| 352 | NRM |  |  | 1550 | 0.331 | 0.6266 | No |
| 353 | CLIC1 |  |  | 1552 | 0.331 | 0.6275 | No |
| 354 | TIMM10 |  |  | 1567 | 0.329 | 0.6248 | No |
| 355 | TMEM237 |  |  | 1576 | 0.328 | 0.6238 | No |
| 356 | PGAM1 |  |  | 1659 | 0.316 | 0.6027 | No |
| 357 | PXMP2 |  |  | 1665 | 0.315 | 0.6024 | No |
| 358 | LSM4 |  |  | 1676 | 0.313 | 0.6008 | No |
| 359 | PSMA3 |  |  | 1692 | 0.312 | 0.5978 | No |
| 360 | VOPP1 |  |  | 1695 | 0.311 | 0.5983 | No |
| 361 | TMX1 |  |  | 1703 | 0.310 | 0.5975 | No |
| 362 | PSMG2 |  |  | 1723 | 0.307 | 0.5934 | No |
| 363 | TMEM14C |  |  | 1739 | 0.306 | 0.5904 | No |
| 364 | WDR54 |  |  | 1750 | 0.304 | 0.5887 | No |
| 365 | ATP5MC1 |  |  | 1769 | 0.302 | 0.5849 | No |
| 366 | OIP5 |  |  | 1789 | 0.299 | 0.5808 | No |
| 367 | AKR7A2 |  |  | 1846 | 0.293 | 0.5667 | No |
| 368 | PRTFDC1 |  |  | 1866 | 0.290 | 0.5625 | No |
| 369 | GLO1 |  |  | 1867 | 0.290 | 0.5635 | No |
| 370 | PPP1CA |  |  | 1954 | 0.279 | 0.5413 | No |
| 371 | MRPL18 |  |  | 1972 | 0.276 | 0.5376 | No |
| 372 | XRCC5 |  |  | 2013 | 0.271 | 0.5277 | No |
| 373 | EEF1B2 |  |  | 2041 | 0.268 | 0.5214 | No |
| 374 | PRADC1 |  |  | 2058 | 0.266 | 0.5179 | No |
| 375 | PGK1 |  |  | 2126 | 0.260 | 0.5008 | No |
| 376 | MXD3 |  |  | 2156 | 0.256 | 0.4938 | No |
| 377 | XRCC6 |  |  | 2180 | 0.254 | 0.4885 | No |
| 378 | GNG4 |  |  | 2702 | -0.316 | 0.3491 | No |
| 379 | CCDC167 |  |  | 2753 | -0.327 | 0.3367 | No |
| 380 | FKBP3 |  |  | 2866 | -0.346 | 0.3077 | No |
| 381 | TCEAL9 |  |  | 2888 | -0.353 | 0.3032 | No |
| 382 | GAMT |  |  | 3145 | -0.413 | 0.2356 | No |
| 383 | NFIC |  |  | 3194 | -0.427 | 0.2241 | No |
| 384 | NME4 |  |  | 3252 | -0.442 | 0.2102 | No |
| 385 | ASRGL1 |  |  | 3362 | -0.483 | 0.1824 | No |
| 386 | METRN |  |  | 3468 | -0.526 | 0.1559 | No |
| 387 | OLFM1 |  |  | 3521 | -0.554 | 0.1437 | No |
| 388 | MCUB |  |  | 3790 | -0.778 | 0.0741 | No |
| 389 | CSRP2 |  |  | 4000 | -1.238 | 0.0219 | No |
| 390 | INAFM1 |  |  | 4001 | -1.245 | 0.0262 | No |
Table: GSEA details [plain text format]

  

Fig 2: DOROSZKO\_U3180MG\_CLUSTER\_3: Random ES distribution      
 Gene set null distribution of ES for **DOROSZKO\_U3180MG\_CLUSTER\_3**
